# Electron tomography of negatively stained complex viruses: application in their diagnosis

**DOI:** 10.1186/1746-1596-4-5

**Published:** 2009-02-10

**Authors:** Jan Mast, Lien Demeestere

**Affiliations:** 1 EM-unit, CODA-CERVA, Groeselenberg 99, Brussels, Belgium

## Abstract

**Background:**

Electron tomographic analysis can be combined with the simple and rapid negative staining technique used in electron microscopy based virus diagnosis.

**Methods:**

Standard negative staining of representative examples of parapoxviruses and paramyxoviruses was combined with electron tomographic analysis.

**Results:**

Digital sectioning of reconstructions of these viruses at a selected height demonstrated the viral ultrastructure in detail, including the characteristic diagnostic features like the surface threads on C-particles of a parapoxvirus and individual glycoproteins and the internal nucleoprotein strand of Newcastle disease virus. For both viruses, deformation and flattening were observed.

**Conclusion:**

The combination of negative staining of complex viruses with electron tomographic analysis, allows visualizing and measuring artifacts typical for negative staining. This approach allows sharp visualisation of structures in a subnanometer-thick plane, avoiding blurring due to superposition which is inherent to TEM. In selected examples, such analyses can improve diagnosis of viral agents.

## Background

Negative staining has been a useful specimen preparation technique for biological and medical electron microscopists for almost 50 years since its introduction by Brenner and Horne as an established method [1]. The technique is based on the embedding of film-supported particles in a matrix of heavy metal stain to increase contrast of electron-lucent particles, and over time numerous modifications and improvements were reported [2]. Because of the simplicity and speed of this technique, one of its major applications is the detection and identification of viruses. Such electron microscopic diagnosis is uniquely suited for the identification of infectious agents in emergent situations because of its speed, and its "open view", i.e. the ability to detect multiple viruses without the need for specific reagents [3].

Virus diagnosis based on morphology is hindered by the limitations of both negative staining and transmission electron microscopy (TEM). Limitations of negative staining, reviewed in detail in [4], include artefacts caused by drying, dehydration and flattening of specimens. Limitations of TEM include the destructive effects of irradiation and blurring of fine ultrastructural details due to superposition of projected features. Hence, the interpretation of micrographs of viruses is more difficult when they are complex and lack symmetry.

At least part of the 'hidden' three-dimensional (3D) information can be retrieved from two-dimensional (2D) projections, like micrographs, by tomographic reconstruction. The mathematical basis for tomographic imaging was already described and formulated in 1917 [5] in the so-called projection-slice theorem. It was however only in the twenty first century that electron tomographic reconstruction became accessible more widely by the integration and computer-control of the goniometric stage, the CCD-camera and the dosimetry of modern TEM. This type of equipment becomes more and more available in laboratories performing diagnostic EM, and data acquisition, alignment and reconstruction software evolves to be more user-friendly.

In this report, we aimed to demonstrate that electron tomographic analysis of negatively stained complex viruses, like paramyxoviruses and parapoxviruses, not only allows visualizing and measuring artifacts like deformation and flattening, but also yields additional and relevant structural information. In selected examples, such analyses can improve diagnosis of viral agents.

## Methods

The seed stock of the Newcastle disease virus (NDV) LaSota strain was obtained from Dr. D.J. Alexander (International Reference Laboratory for Newcastle disease, Weybridge, United Kingdom) and propagated in the allantoic cavity of 9- to 11-day old SPF embryonated chicken eggs as described [6]. Parapoxvirus (Orf virus) was obtained from a sheep manifesting pustular lesions on its oral mucosa. A crude virus suspension was obtained by mechanical extraction of a crust using a pestle and a grinder tube. To verify reconstructions polystyrene beads (mean diameter 112 nm, Agar Scientific, Stansted, UK) were added to certain samples as internal controls.

A standard protocol for negative staining was applied to these virus suspensions. Pioloform- and carbon-coated 400 mesh copper grids (Agar Scientific) were pre-treated with 1% Alcian Blue (Fluka, Buchs, Switzerland) to increase hydrophilicity and rinsed 5-times with water. The grids were deposited on a drop of viral suspension for 10 minutes and rinsed two times with water. For NDV, the virus-coated grids were incubated in addition for 1 minute with a suspension of 10-nm gold particles (goat-anti-mouse IgG 10 nm, Aurion, Wageningen, Netherlands) as fiducial markers for image alignment. Afterwards, the grids were stained for 10 seconds on a drop of 2% uranyl acetate (Agar Scientific) or 1% potassium phosphotungstate (pH 7.2, EMS, Hatfield, US).

Specimens were mounted in a dedicated holder (Model 2020 from Fischione instruments, UA) and analyzed using a Technai Spirit TEM (FEI company, Eindhoven, The Netherlands) with a BioTwin lens configuration and a Lab6-filament operating at an acceleration voltage of 120 kV.

Series of micrographs (tilt-series) were recorded semi-automatically assisted by the Explore 3D tomography-module of the microscope control software over a tilt range of ± 70°, or highest angle possible, at intervals of 1.5°. Shift and focus changes were corrected at every interval. Images were acquired with a 4*4 k Eagle CCD-camera (FEI) at a magnification 49,000 times and a corresponding pixel sizes of 0.22 nm. The tilt series were aligned using the Inspect 3D software, version 2.5 (FEI). Tilt series of Orf virus were aligned by rounds of cross correlation. Tilt series of NDV were aligned by combining alignment based on two rounds of cross correlation with feature tracking based on 6 or more gold beads. Reconstructions using iterations of the SIRT algorithm were superior to reconstructions based on weighted back projection. Visualization was done using the AMIRA software (Mercury, France). The processing of a sample by this method takes about 12 hours.

The density map of the F-glycoprotein of Newcastle disease virus, as obtained by crystallography (RCSB protein databank entry 1G5G [7]), was represented using the UCSF chimera software [8].

## Results

### Electron tomography of a parapoxvirus

**Figure 1:**
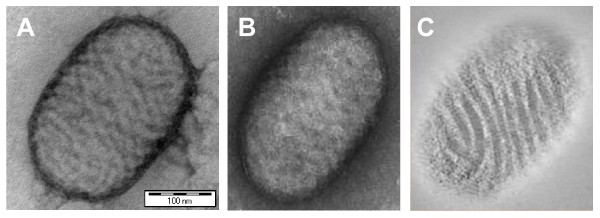
**Negative staining and electron tomographic analysis of Orf parapoxvirus**. Figure 1A and B represent micrographs of uranyl acetate-stained particles of M-type and C-type Orf parapoxvirus characterised by obvious and hardly visible surface threads, respectively. Figure 1C is a 44-nm thick digital section taken along the Z-axis of the tomogram of the C-particle shown in B. A, B and C are represented with the same scale. Bar: 100 nm.

Negatively stained virions of Orf virus, representing the parapox genus, are ellipsoid-shaped and measure approximately 250 by 150 nm. A typical diagnostic feature are the surface threads that, because both sides of the virus are seen in the microscope, give a criss-cross appearance (Figure 1A). Depending on the integrity of the virions and associated penetration of stain, threads become less (Figure 1B) or no longer visible, although the surface undulation can be recognised as the surface threads seen in profile. In analogy with the nomenclature of orthopoxviruses, the particles shown in A and B are referred to as mulberry or M-type and capsule or C-type particles, respectively, although no clear internal structures or lateral bodies as seen with orthopoxviruses, can be observed in a C-particle of parapoxviruses. By electron tomography, 3D-reconstructions of negatively stained virions of Orf virus were made. In digital slices of such reconstruction that are taken at the level of the particle envelope along the Z-axis of the tomogram, the surface threads can be demonstrated easily, even in C-particles (Figure 1C, Additional file 1). Their semi-parallel orientation indicates that the threads, instead of being randomly arranged, form a more or less regular spiral which could be a continuous structure. The animated version of the surface-rendered 3D-reconstruction of this virion shown in Additional file 2 illustrates this further. This animation and the snapshots of a slightly modified 3D-reconstruction of the same virion shown in Additional file 3 demonstrate a strong flattening of the virion. Very similar results were obtained in eight independent tomograms. Reconstructions of polystyrene beads that were added to the samples as internal controls showed no deformation and the expected spherical symmetry for all examined beads (not shown), confirming the validity of the reconstructions.

### Electron tomography of a paramyxovirus

**Figure 2:**
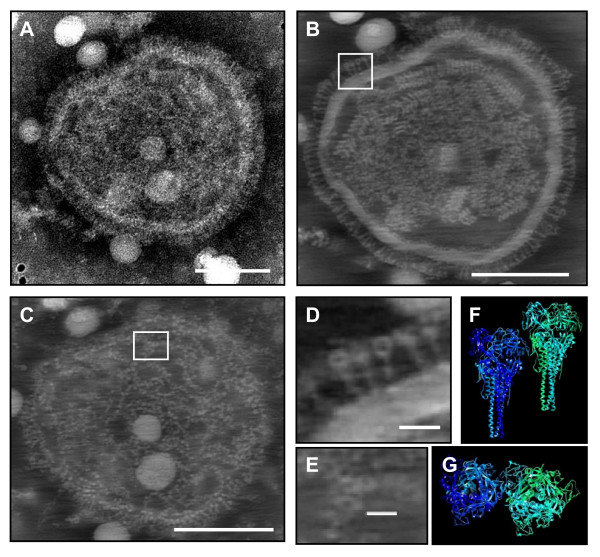
**Negative staining and electron tomographic analysis of Newcastle disease paramyxovirus**. Figure 2A represents a micrograph of a PTA-stained virion. Figure 2B and C are 44-nm thick digital sections taken along the Z-axis of the tomogram of the virion shown in Figure 2A, taken in the central part (B) and at the level of the envelope (C). Figure 2D and Figure 2E represent the inserts in Figure 2B and Figure 2C, respectively, at higher magnification. Figure 2F and Figure 2G represent side and top views of the 3D ribbon-model of the density map of the F-glycoprotein of Newcastle disease virus. Bar A, B and C: 100 nm, Bar D: 10 nm, Bar E: 8 nm.

Figure 2A shows a negatively stained virion of Newcastle disease virus. It is characterised by a peripheral fringe of glycoprotein spikes that is so dense that individual spikes are not clearly visible. A second feature characteristic for paramyxoviruses, namely the internal ribonucleoprotein helix can in this case only be suspected. 3D-reconstructions of negatively stained virions of NDV were made by electron tomography. These revealed a strong flattening of the virions, with particle dimensions in the Z-direction typically less than 50 nm (not shown). In 0.44-nm-thick digital slices of such reconstructed virions, individual glycoproteins of the fringe could be revealed readily (Figure 2B, Figure 2C and Additional file 4). Fine ultrastructural details of specific glycoproteins like the head, the neck and the stalk region were demonstrated (Figure 2C and Figure 2D). These glycoproteins are 17 nm tall. The diameter of the heads, visible in the section shown in figure 2C as circles is approximately 6 nm, while the diameter of the stalks, visible as small white dots, is approximately 2 nm.

The silhouette of these glycoproteins strongly resembles the silhouette of the 3D model obtained from the density map of the F-glycoprotein of NDV. In digital sections of the central part of the virions (Figure 2B and Additional file 4), and particularly in stain-penetrated virions, the ribonucleoprotein was shown centrally as a fragmented strand of helical structures of variable length, a mean diameter of 17 nm, a central canal of approximately 5 nm and a pitch of 1 rotation per 5 nm. The ribonucleoprotein was surrounded by an electron-lucent envelope with the same thickness as the fringe of glycoproteins (14–18 nm thick). Very similar results were obtained in twenty independent tomograms.

**Figure 3:**
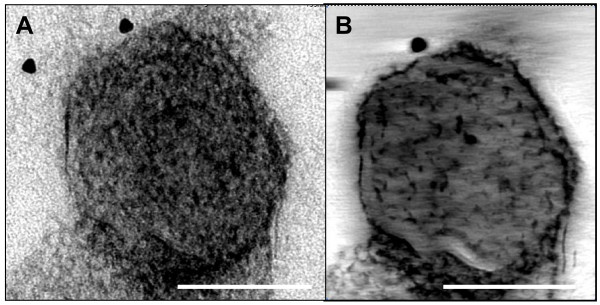
**Negative staining and electron tomographic analysis of a suspected feature**. Figure 3A represents a micrograph of a negatively stained feature that, based on its size and general aspect, can be mistaken for a virion of a paramyxovirus. Figure 3B is a central digital section of 44 nm thick along the Z-axis of the tomogram of the feature shown in Figure 3A. Bar A, B: 100 nm.

Figure 3A shows a representative negatively stained particle that, based on its size and general appearance, could be mistaken for a virion of a paramyxovirus virion. It originates from a faeces sample obtained from a calf where a viral aetiology was suspected. In this case, digital sections in the centre of the particle revealed no proteins at the surface of the membrane and an unstructured content. Any of the diagnostic features shown in Figure 3B could be demonstrated, excluding a paramyxovirus as a possible etiological agent.

## Discussion

The electron tomographic reconstructions of negatively stained virions of parapoxviruses and paramyxoviruses have revealed ultrastructural details that in the standard micrographs can not or hardly be distinguished. Digital sectioning of the reconstructions at a selected height allowed sharp visualisation of structures in a subnanometer-thick plane, avoiding blurring due to superposition which is inherent to TEM.

For Orf parapoxvirus the characteristic surface threads were clearly demonstrated in C-type particles where only surface undulation suggests the presence of threads seen in profile. Their semi-parallel orientation supports the suggestion of Madely and Field [9] that they spiral continuously around the virions. In most practical cases microscope operators will search longer for typical thread-covered M-particles, if necessary making another preparation to come to a definitive diagnosis [9]. In specific cases, like differential diagnoses with foot-and-mouth disease virus and suspicions of human orthopoxvirus, samples are for biosecurity reasons treated with high concentrations of inactivation agents, damaging the integrity viral envelopes. Further, the suboptimal taking and storage of clinical samples can, particularly in skin lesions, negatively affect virus morphology. In such samples only stain-penetrated C-particles are found, and demonstration of surface threads by electron tomography will be useful to come to a diagnosis, the more since the time investment is entirely justified in these cases.

For NDV, individual glycoproteins were shown both on the periphery of virions and on the lower an upper parts of the viral envelope. Details of these glycoproteins like the cup-shaped head and a 2-nm-wide stalk were shown, and their sizes and silhouettes corresponded seamlessly with the sizes and silhouettes of the crystallography-based model of the NDV fusion protein [10]. Although some variation can be seen among glycoproteins, differentiation between the haemagglutinine-neuraminidase (HN) and the fusion (F) glycoproteins will require further work. One complication is that a high resolution density map obtained by crystallography, which could serve as a reference, is not available for the entire HN glycoprotein. The 3D model described in [11] comprises only the head and not the entire protein. Other diagnostic characteristics like the ribonucleoprotein and the envelope were visualised and measured. The 14 to 18 nm wide envelope suggests tight association of (matrix) protein with the cell-derived phospholipid membrane.

Because of their pleiomorphic appearance and densely packed fringe, it can be difficult to distinguish paramyxoviruses from mycoplasms [9] and/or membranous cell debris such as that from mitochondria that can have knobs and resemble the spiked membrane viruses [12]. Moreover, paramyxoviruses can be particularly difficult to identify in negatively stained specimens and a confident identification is often only made if a free nucleocapsid is observed. Figure 3 clearly demonstrated that in such cases, electron tomographical reconstruction of negatively stained particles can eliminate possible doubt.

The rapid and simple negative staining procedure used here and in most diagnostic laboratories lacks the near-nature-state fixation of cryoelectron microscopy and results in structural perturbations. Electron tomographic reconstructions of both negatively paramyxoviruses and parapoxviruses demonstrated extensive flattening, while the fragmentation of the ribonucleoprotein strand of the paramyxovirus most likely results from shearing forces during drying. This approach can thus be used efficiently to objectify the artefacts induced by negative staining, reviewed in [4], which may be important to explain the large variations in size and morphology of identical viruses reported in literature. Cryoelectron tomography of unstained specimens avoids these artefacts and is the method of choice for the visualisation of structures of complex viruses and their components [13]. This methodology requires however access to a modern top-of-the-range electron microscope, which is limited. Cryonegative staining [14] combined with tomographic 3D image reconstruction could be a useful alternative to collect high resolution data on a 120 kV cryoelectron microscope [2]. Regrettably, this technique also requires high concentrations of relatively pure particles which are not compatible with the needs of diagnostic EM.

Despite these shortcomings, the presented examples illustrate that the simple negative staining procedure shares certain advantages with cryonegative staining over unstained vitreous specimens. The combination of enhanced contrast and samples being less susceptible to radiation damage [4,15] allow extensive data collection and improve cross correlation-based alignment of the tilt series. This implies in practice that for negatively stained samples a tilt series can be recorded for any individual particle of interest without substantially altering the microscope settings, provided that the grid is mounted in a holder allowing at high tilt angles and that the specimen is not situated close to a grid bar. In digital sections along the Z-axis of a tomogram, blurring of fine ultrastructural details due to superposition of projected features is reduced and, based on the observed ultrastructural details (Figure 2D), a resolution of 2 nm or lower can be expected. The relatively long processing time of 12 hours can be reduced to less than 3 hours if the newer 3.0 version of Inspect 3D alignment and reconstruction software and a dedicated workstation are used.

In conclusion, the combination of negative staining of complex viruses with electron tomographic analysis, allows visualizing and measuring artifacts typical for negative staining. This approach allows sharp visualisation of structures in a subnanometer-thick plane, avoiding blurring due to superposition which is inherent to TEM. In selected examples, such analyses can improve diagnosis of viral agents.

## Competing interests

The authors declare that they have no competing interests.

## Authors' contributions

Both authors have contributed equally to the preparation of the manuscript. Both authors read and approved the final manuscript.

## Supplementary Material

Additional file 1 **Tomogram of a parapoxvirus**. The video shows a negatively stained virion of the Orf parapoxvirus, a section of which is shown in Figure 1C. Each frame of the movie corresponds to a 0.44 nm-thick digital section through the tomogram moving along the tomographic Z-axis (Total Depth nm). Observe the semi-parallel orientation of the surface threads. Click here for file

Additional file 2 **Surface rendered view of a parapoxvirus.** The video shows a surface rendered view of the parapoxvirus particle seen in additional file 1. Figure 1C represents a section of this movie. Observe the surface threads that, instead of being randomly arranged, form a more or less regular spiral Click here for file

Additional file 3 **False-colored 3D-rendering of a C-particle of a parapoxvirus**. This figure shows snapshots of a false-colored 3D-rendering of the C-particles shown along the tomographic Y-axis (A) and Z-axis (B) se-colored rendering surface rendered view of the parapoxvirus particle seen in additional file 1. Figure 1C represents a section of this movie. Click here for file

Additional file 4 **Tomogram of Newcastle disease virus**. The video shows a negatively stained virion of the Newcastle disease LaSota strain, a section of which is shown in Figure 2B. Each frame of the movie corresponds to a 0.44 nm-thick digital section through the tomogram moving along the tomographic Z-axis. Click here for file
