# Supplementary figures and images for: Electron tomography of negatively stained complex viruses: application in their diagnosis
Source: Diagn Pathol. 2009 Feb 10;4:5. doi: 10.1186/1746-1596-4-5 (PMC2649040; doi:10.1186/1746-1596-4-5)

## Slide 1
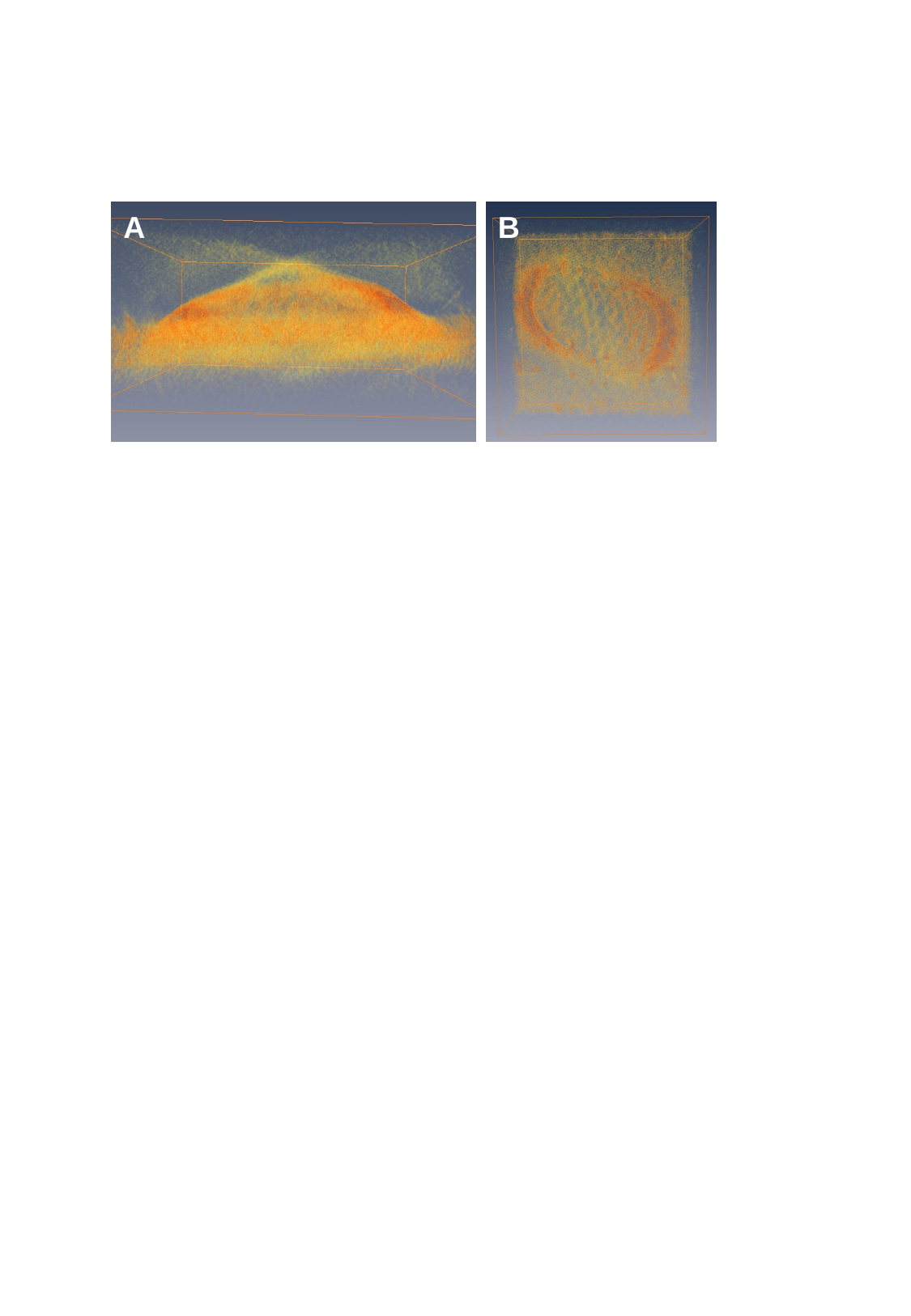

A
B

Supplement: Additional file 3 — False-colored 3D-rendering of a C-particle of a parapoxvirus. This figure shows snapshots of a false-colored 3D-rendering of the C-particles shown along the tomographic Y-axis (A) and Z-axis (B) se-colored rendering surface rendered view of the parapoxvirus particle seen in additional file 1. Figure 1C represents a section of this movie. [file 1746-1596-4-5-S3.ppt]
